# Loss of heterozygosity on chromosome 16 in sporadic Wilms' tumour.

**DOI:** 10.1038/bjc.1998.651

**Published:** 1998-11

**Authors:** R. G. Grundy, J. Pritchard, P. Scambler, J. K. Cowell

**Affiliations:** Haematology Oncology Unit, Institute of Child Health, London, UK.

## Abstract

**Images:**


British Journal of Cancer (1998) 78(9), 1181–1187
© 1998 Cancer Research Campaign

### Loss of heterozygosity on chromosome 16 in sporadic Wilms' tumour

**RG Grundy[1]*, J Pritchard[1,2], P Scambler[3] and JK Cowell[1†]**

[1]Haematology Oncology Unit, Institute of Child Health, 30 Guilford Street, London WC1N 1EH; [2]Department of Haematology/Oncology, Hospitals for Sick Children, Great Ormond Street, London WC1; [3]Molecular Medicine Unit, Institute of Child Health, 30 Guilford Street, London WC1N 1EH, UK

**Summary** To establish whether loss of heterozygosity (LOH) for chromosome 16q in Wilms' tumours confers an adverse prognosis, DNA from 40 Wilms' tumour/normal pairs were analysed using highly polymorphic microsatellite markers along the length of 16q. Fifteen per cent of tumours showed LOH for 16q. Although the common region of allele loss spanned the 16q24–qter region, a second distinct region of LOH was identified in 16q21. Five out of six tumours showing LOH were either (1) high stage or (2) low stage with unfavourable histology. In addition, there was a higher mortality rate in patients showing LOH for 16q than those that did not. These data strongly support the suggestion that LOH for 16q is associated with an adverse prognosis.

**Keywords:** Wilms' tumour; loss of heterozygosity; chromosome 16; prognosis

The overall cure rate for Wilms' tumour is now 85%, a consequence of consistent improvement in treatment strategies (D'Angio et al, 1989; Pritchard et al, 1995). Refinement of therapy has been based on the traditional prognostic factors of tumour histology and the clinicopathological stage. However, these factors have their limitations. For example, histological 'anaplasia' is found in only 5% of Wilms' tumours and is partly stage dependent, conferring an adverse prognosis in stage 2, 3 and 4 tumours, but not in stage 1 disease (D'Angio et al, 1981). The addition of doxorubicin to vincristine and actinomycin D in the National Wilms' Tumour Study (NWTS-3) improved the relapse-free survival by 10% and the overall survival by 5% in patients with 'favourable histology' stage 3 disease (D'Angio et al, 1989). In other words, among children with stage 3 disease, there is a subset of patients that benefit from the addition of doxorubicin, although the majority may still be cured without using this drug. This improvement in survival is, however, achieved at a price, as the use of doxorubicin is associated with considerable cardiac late effects (Goorin et al, 1990; Sorenson et al, 1995). Clearly, if we are to continue to improve the outcome of patients with Wilms' tumour, while minimizing the late effects of curative therapy, new prognostic factors are required.

Cytogenetic abnormalities of chromosome 16 have been reported in 30% of Wilms' tumour and predominantly involve the long arm (Slater et al, 1992; Austruy et al, 1995). Two regions of interest have been identified from these studies, a proximal locus at 16q11–13 and a more distal region involving 16q21–24–qter (Slater et al, 1992). An identical translocation, der (16)t(1;16)(q21;q13), has also been reported in four Wilms' tumours (Solis et al, 1988; Wang-Wuu et al, 1990), and a der (16)t(1;16)(q12;q12) was reported by Kaneko et al (1991). Tumour-specific allelic loss has been thought to represent the second hit, resulting in the inactivation of a tumour-suppressor gene and can be detected by LOH analysis (Knudson and Strong, 1972; Cavenee et al, 1983). Molecular studies have found LOH for polymorphic markers on 16q in 10–25% of Wilms' tumours (Coppes et al, 1992; Maw et al, 1992; Grundy et al, 1994; Austruy et al, 1995; Redeker et al 1996). Using a panel of informative markers for the region 16q12.2–qter, Maw et al (1992) were the first to demonstrate LOH for 16q in 9 (20%) of 45 informative patients by Southern blot analysis. A larger follow-on study, as part of NWTS-4, involving 232 patients, found a similar frequency of LOH at 17%, but used only polymorphic markers that mapped to the distal 16q22–qter region (Grundy et al, 1994). In the study by Coppes et al (1992), using only two polymorphic markers mapping to 16q22.2 and 16q24.3, LOH was identified in 20% of tumours. The most recent study, albeit in a relatively small group of patients (Austruy et al, 1995), found the highest level of LOH (25%) using a large panel of restriction length polymorphisms and microsatellite probes. Furthermore, LOH for 16q has been associated with a worse outcome because these patients have a relapse rate 3.3 times higher than those with tumours retaining heterozygosity for 16q (Grundy et al 1994).

Because cytogenetic and molecular studies of Wilms' tumours clearly suggest a role for genes on 16q in the molecular pathology of this tumour, we have undertaken LOH analysis for 16q markers using a large, well-characterized series of sporadic Wilms' tumours. Our results clearly show that LOH is relatively frequent in Wilms' tumours, especially in patients with an adverse outcome.

### MATERIALS AND METHODS

DNA was prepared from tumour tissue and lymphocytes using standard phenol–chloroform extraction procedures described by Wadey et al (1990). The optimal polymerase chain reaction (PCR) conditions

*Received 24 December 1997*
*Revised 31 March 1998*
*Accepted 7 April 1998*

*Correspondence to:* RG Grundy

**Present address:* Department of Oncology, The Birmingham Children's Hospital, Steelhouse Lane, Birmingham B4 6NH.

†*Present address:* Department of Neurosciences, Lerner Research Institute, Cleveland Clinic Foundation, 9500 Euclid Avenue, Cleveland, Ohio 44195.

**Table 1** Microsatelite repeat primer pairs for chromosome 16q LOH analysis

| Locus symbol | Physical location | Heterozygosity | CA strand primer | GT strand primer | Size range (bp) | [$Mg^{2+}$] (mmol) | Annealing temperature |
|---|---|---|---|---|---|---|---|
| D16S519 | 16p12 | 0.81 | AGCTTACCAGTCTCACAGGG | AAACCATGCTTGTCTAGCC | 135–157 | 2.0 | 58°C |
| D16S419 | 16q12–13 | 0.76 | ATTTTAAAGGAATGTAAAGNACACA | GACGTTAGACCAGGAGTCAG | 146–164 | 1.5 | 56°C |
| D16S514 | 16q21 | 0.82 | CTATCCACTCACTTTCCAGG | TCCCACTGATCATCTTCTC | 117–129 | 1.5 | 56°C |
| D16S512 | 16q22.1 | 0.82 | TGAGAGCCAAATAAATAAATGG | ATAAGCCACTGCGCCCAT | 201–211 | 2.0 | 58°C |
| D16S518 | 16q23.1–24.2 | 0.83 | GGCCTTTTGGCAGTCA | ACCTTGGCCTCCACAC | 272–290 | 2.0 | 57°C |
| D16S520 | 16q24 | 0.84 | GCTTAGTCATACGAGCGG | TCCACAGCCATGTAAACC | 181–197 | 2.0 | 58°C |
| D16S413 | 16q24.3 | 0.84 | ACTCCAGCCCGAGTAA | GGTCACAGGTGGGTTC | 131–149 | 1.5 | 56°C[a] |

[a]1% DMSO.

**Table 2** Wilms' tumour samples used in WT1 mutation analysis

| No. | Tumour sample (GOS no.) | Disease stage | Histopathology | Status | (LOH) |
|---|---|---|---|---|---|
| 1 | 249 | 1 | Mononorphous epithelial | Alive | (No) |
| 2 | 145 | 3 | FH[a] | Alive | (No) |
| 3 | 16 | 3 | FH | Alive | (No) |
| 4 | 54 | 3 | FH | Alive | (No) |
| 5 | 55 | 2 | FH | Alive | (No) |
| 6 | 101 | 1 | UH | Relapsed/alive | (Yes) |
| 7 | 185 | 1 | FH | Alive | (No) |
| 8 | 120 | 4 | FH | Alive | (No) |
| 9 | 132 | 1 | FH | Alive | (No) |
| 10 | 90 | 1 | FH | Alive | (No) |
| 11 | 244 | 3 | FH | Alive | (No) |
| 12 | 219 | 1 | FH | Alive | (No) |
| 13 | 129 | 3 | FH | Alive | (No) |
| 14 | 231 | Bilateral | FH | Alive | (No) |
| 15 | 100 | 2 | FH | Alive | (No) |
| 16 | 207 | 1 | FH | Alive | (No) |
| 17 | 44 | 4 | FH | Alive | (No) |
| 18 | 89 | 3 | FH | Alive | (No) |
| 19 | 135 | 3 | FH | Alive | (No) |
| 20 | 146 | 1 | FH | Alive | (No) |
| 21 | 218 | Bilateral | FH | Alive | (No) |
| 22 | 126 | 3 | FH | Alive | (No) |
| 23 | 51 | 3 | FH | Alive | (No) |
| 24 | 360 | 1 | FH | Alive | (No) |
| 25 | 270 | 4 | FH | Alive | (No) |
| 26 | 399 | 1 | Mononorphous epithelial | Alive | (No) |
| 27 | 407 | 3 | UH | Relapsed/died | (No) |
| 28 | 446 | 1 | FH | Alive | (No) |
| 29 | 206 | 4 | FH | Alive | (Yes) |
| 30 | 234 | 3 | FH | Alive | (No) |
| 31 | 169 | 4 | FH | Alive | (Yes) |
| 32 | 439 | 3 | FH | Alive | (No) |
| 33 | 178 | 1 | FH | Alive | (No) |
| 34 | 66 | Bilateral | FH | Alive | (Yes) |
| 35 | 542 | 3 | FH | Relapsed/died | (Yes) |
| 36 | 505 | Bilateral | FH | Alive | (No) |
| 37 | 119 | 1 | FH | Alive | (No) |
| 38 | 96 | 4 | FH | Alive | (No) |
| 39 | 198 | Bilateral | FH | Relapsed/died | (Yes) |
| 40 | 358 | 4 | FH | Relapsed/alive | (No) |

[a]In the absence of anaplastic nuclear changes the term favourable histology (FH) is used because of the generally good outcome for these patients.

for each of the microsatellite primer pairs are detailed in Table 1. The CA strand primer was radiolabelled with gamma $^{32}P$ (0.4 µCi) using polynucleotide kinase (NBL). The PCR was performed using a Biometra thermal cycler in a reaction volume of 10 µl containing 50–100 ng of tumour or constitutional DNA. dNTPs at a final concentration of 0.2 mM, 1.5–2 mmol of magnesium chloride, 1–2 pmol of labelled and unlabelled primer, *Taq* DNA polymerase 0.5 U and 1X Buffer (Bioline). The step cycle file comprised 3 min of denaturation at 94°C, 30 cycles of 94°C for 30 s, annealing at the appropriate temperature for 30 s and extension at 72°C for 30 s. The annealing temperature varied with each primer system (Table 1). On completion, 6 µl of stop solution (Gibco BRL) was added to each sample and

© Cancer Research Campaign 1998

**Figure 1** Microsatellite analysis at the various chromosome 16-specific loci (left) for each of the six tumours showing LOH. In all cases the PCR products from the normal sample is shown on the left of each pair and the tumour samples is on the right. Where loss of alleles was demonstrated in the tumour, this is indicated by the arrows denatured at 95°C for 3 min. An aliquot of 3 µl of the resultant solution was loaded onto a 6% denaturing polyacrylamide gel. The gel was run at 60 W constant power in 1 × Tris acetate EDTA buffer; the length of time depended on the fragment size. Gels were then fixed in 10% methanol/10% acetic acid before drying and exposure to XAR-5 film (Kodak) for 12–72 h without an intensifying screen.

Tumour samples were collected between May 1983 and May 1990. All patients referred to the Hospital for Sick Children, Great Ormond Street, with a histopathological diagnosis of Wilms' tumour were treated on sequential United Kingdom Children's Cancer Study Group protocols UKW1 and UKW2 (Pritchard et al, 1995; CD Mitchell, personnal communication). All children received uniform treatment as per the protocol and have been followed up for at least 7 years.

### RESULTS

In all, 40 Wilms' tumours from patients who presented to the Hospitals for Sick Children, London, were available for LOH analysis. Following the clinical diagnosis of a 'Wilms' tumour', the patients underwent nephrectomy and DNA was prepared from the tumours. The clinicopathological details of these 40 Wilms' tumours are summarized in Table 2. Two of the tumours analysed in this study were also of a very unusual histological subtype, showing monomorphous epithelial changes. These tumours arose in a brother and sister; their mother had been cured of a Wilms' tumour by nephrectomy 20 years previously. In view of this history of familial Wilms' tumour, GOS 149 and 399 alone cannot be classified as sporadic Wilms' tumours, but were still analysed for LOH on 16q. All of the microsatellite polymorphic markers tested had a frequency of heterozygosity above 0.73 and all 40 patients were heterozygous at three or more of the ten polymorphic loci tested. The physical location of the polymorphic microsatellite markers was obtained from Kozman et al (1995) and Doggett et al (1995) and from the Genome database (Gyapay et al, 1994).

### Clinicopathological details of the tumours

Of the 40 patients analysed in this study, 13 had stage 1 disease, two stage 2, 13 stage 3, seven stage 4 and five had bilateral disease. Thirty-eight patients had 'favourable' histology (FH) tumours and two had 'unfavourable' histological features (UH). Five children relapsed and three of these patients died of disease, with two being cured by second-line therapy.

### Loss of heterozygosity on chromosome 16

Constitutional and tumour DNA from 40 patients were used to investigate the frequency of LOH along the long arm of chromosome 16, using seven polymorphic microsatellite markers (D16S413, D16S 520, D16S 518, D16S 512, D16S 514, D16S 419 and D16S519). The physical location of the polymorphic microsatellite markers is shown in Table 1 and was determined from the genome database and current literature (Gyapay et al 1994; Doggett et al 1996). All polymorphic microsatellite markers had a heterozygosity frequency above 0.75 (see Table 1).

Six (15%) of 40 informative Wilms' tumours – GOS 66, GOS 101, GOS 169, GOS 198, GOS 206, GOS 542 – showed LOH for markers on 16q. In these six cases there was loss of one allele in the tumour compared with constitutional DNA. No homozygous deletions were detected. The results of the LOH analysis are shown in Figure 1 and diagrammatically in Figure 2. In two of the

Figure 2 ???

© Cancer Research Campaign 1998

six tumours showing LOH – GOS 101 and 169 – demonstrable allelic loss was restricted to the 16q23–qter region. The other four tumours, GOS 66, GOS 198, GOS 206 and GOS 542, also showed LOH for the D16S520 locus, making this the single, consistent region lost in all tumours. The more distal D16S413 locus shows LOH in three tumours, but whether this region is also involved in tumours GOS 66, GOS 198 and GOS 542 cannot be determined because these tumours were not informative at this locus. None of the tumours experienced LOH distal to the D16S514 locus, which showed LOH in three tumours and possibly in GOS 206, which was uninformative at this locus. Tumours GOS 66, GOS 198 and also GOS 206 all show LOH for at least one locus proximal to D16S518 but, because some of the interstitial markers were constitutionally homozygous in these tumours, it could not be demonstrated whether any loci within the D16S514–D16S520 interval had retained heterozygosity. From the analysis of these five tumours, therefore, the results are consistent with LOH being initiated at a defined point along 16q, which is different in different tumours, and extending distally to 16qter. Tumour GOS 542, however, shows LOH or constitutional homozygosity for the 16q23.2–qter region, as well as LOH for D16S514 in 16q21. Heterozygosity is retained at the intervening D16S512 locus. In this tumour, therefore, there is demonstrable discontinuity in LOH, thereby defining a second distinct region that potentially contains a tumour-supressor gene(s) (Figure 2). The most proximal marker tested was D16S519, which mapped to 16p12. This marker was informative in all tumours except GOS198 and, in all of the tumours, both alleles were retained (Figure 1). Thus, from the summary shown in Figure 2, two regions of distinct LOH can clearly be identified.

### Clinical outcome and LOH for 16q

Three of the 40 patients with Wilms' tumour in this series, GOS 198, 407 and 542, died of disease and the tumours from two of these, GOS 198 and 542, showed LOH for markers on 16q. Two other patients, GOS 101 and 358, relapsed but were cured by second-line therapy (see Table 2). One of these recurrent tumours, GOS101, also showed LOH for 16q. Although this tumour was stage 1 by clinicopathological staging, it had unfavourable histological features. GOS 407 was a stage 3 tumour with unfavourable histological features and this patient died 1 month after relapse. There was no evidence for LOH for 16q markers in this tumour. Overall, therefore, two of the three patients who died of the disease had LOH for 16q. The only patient with stage 1 disease, who relapsed, also showed LOH for this region (Table 2). These observations demonstrate the tendency for LOH to occur in higher stage disease or with unfavourable histological features. Furthermore, three out of five patients that relapsed, also had LOH for 16q. Even though the patient numbers are small, and because this was a retrospective study using a selected series of tumours, these results still provide strong evidence for a trend towards worse outcome in patients with LOH for 16q.

### DISCUSSION

We have performed LOH analysis in a series of 40 patients with sporadic Wilms' tumour to investigate whether allelic loss on the long arm of chromosome 16 is associated with poor prognosis. Our study found tumour-specific LOH for markers on 16q in 6 (15%) of 40 tumours. Although the frequency of LOH is slightly lower than in other studies (Coppes et al, 1992; Maw et al, 1992; Grundy et al 1994; Austruy et al, 1995), this probably simply reflects minor statistical variation as a result of (1) the relative numbers of tumours used in different studies, (2) the number of specific markers used and (3) the means of detecting the polymorphisms. The overall mean frequency of LOH is approximately 20%. It is also possible, however, that minor differences in the frequency of LOH between studies may reflect genetic differences between different racial groups.

The study by Maw et al (1992) provided molecular evidence for the existence of two discrete regions of LOH on 16q. Region 16q13 was implicated by the discovery of an interstitial deletion between 16q13 and 16q21 in a single tumour. A second tumour had an interstitial deletion, but the extent of the deletion could not be defined because not all of the probes that were used between 16q11.2 and q21 were informative. In five other tumours in this study, LOH was restricted to the 16q22–qter region (Maw et al, 1992). From our analysis it was also possible to establish, unequivocally, that two distinct regions of 16q show LOH in Wilms' tumorigenesis. Clearly LOH for the 16q24–qter region was the most consistent finding but one of the six tumours showed LOH that was restricted to the 16q21 region. Tumours from several patients showing larger regions of LOH were, unfortunately, constitutionally homozygous at critical loci, and so whether any of the other tumours in fact showed 16q21-specific LOH could not be determined (Maw et al, 1992). Importantly, none of these six tumours showed LOH extending to 16q13, which means that, in combination with previous studies, the proximal site of any potential tumour-suppressor gene is restricted to 16q21. In two other studies (Grundy et al, 1994; Coppes et al, 1992), only polymorphic markers that mapped to the telomeric region of 16q were used and so cannot confirm the location of a putative Wilms' tumour (WT) gene in 16q21. Newsham et al (1995), on the other hand, concentrated on the 16q13 region and found that 20% of their panel of 26 tumours showed LOH for the 16q13–21 region. No markers mapping distal to 16q22.1 were used in this study, however, and so it was not determined whether LOH extended all the way to 16qter. Recently, Austruy et al (1995) used a number of markers distributed along the length of 16q and identified LOH in 7/25 tumours. The region of allelic loss extended from 16qter to D16S419 in 16q12–13, which is consistent with our study. Taken together, therefore, LOH studies for chromosome 16 in Wilms' tumour clearly define the 16q24–qter region as the most common site of allelic loss. The evidence for a tumour-supressor gene in 16q21 is still based on only a limited number of observations, which may indicate that genes in these regions are less critical in establishing the tumour phenotype. Interestingly, the der (16) t(1;16) (q21;q13) cytogenetic abnormality was noted in 10% of those tumours that could be karyotyped successfully (Matthew et al, 1996). All of these tumours were of favourable histology and the cytogenetic abnormality did not confer an adverse prognosis (Matthew et al, 1996). Whether the breakpoint in this tumour inactivates a critical tumour-supressor gene has not been determined, but if this is the case then loss of function of this particular gene does not lead to adverse outcome.

From our studies, a clear association between LOH on 16q and higher stage/worse outcome tumours was apparent. Overall, therefore, two of the three patients who died of the disease in this series had LOH for 16q. The only patient with stage 1 disease who relapsed also showed LOH for this region, demonstrating a tendency for LOH to occur in higher stage disease or predict poor

© Cancer Research Campaign 1998

prognosis in patients with stage 1 disease. Furthermore, all but one of the tumours with LOH for 16q markers were stage 3/4 or had bilateral disease at diagnosis. The remaining (sixth) tumour with LOH for 16q markers (GOS101) was stage 1 at diagnosis but had unfavourable histological features (anaplasia). This tumour recurred, but, following second-line treatment, the child is alive with no detectable disease. Although the presence of anaplasia confers an adverse prognostic sign, it is stage related, and does not significantly alter the outcome of patients with stage 1 disease (D'Angio, 1989). Given the observation of Grundy et al (1994), who noted that LOH on 16q was associated with adverse outcome, irrespective of the stage or histological features of the tumour, it is likely that the molecular alteration in GOS 101 is more significant than the presence of anaplasia. Austruy et al (1995) detected LOH for 16q markers in 7 of 25 Wilms' tumours. Two of these were considered to be stage 1 at diagnosis and in both cases the tumour recurred; one patient was subsequently cured, but the other died of disease, again supporting the hypothesis that allelic loss on 16q represents an adverse prognostic marker, irrespective of the disease stage.

Whether the 16q locus will prove to be Wilms' tumour specific is still not clear at this time. A number of other tumour types have been found to exhibit LOH from 16q, usually in addition to a number of other chromosomal abnormalities. The region of allelic loss in a number of different tumour types also appears to involve 16q22–qter (Mugneret et al, 1988; Sato et al, 1990; Tsuda et al, 1990; Bergerheim et al, 1991; Fujimori et al, 1991; Thomas et al, 1991; Douglass et al, 1990; Dorion-Bonnet, et al 1995), the same as that involved in Wilms' tumour, although still a relatively large region.

Nephroblastomatosis is considered by some to be a precursor lesion for Wilms' tumour based on (a) histopathological analysis (Beckwith et al, 1990) and (b) the discovery of WT1 mutations in two patients with so-called nephrogenic rests (Park et al, 1993). LOH for markers in 16q13–qter has also been reported in one case of nephroblastomatosis (Austruy et al, 1995). Although interesting, this observation conflicts with the hypothesis that genes on chromosome 16q are implicated in tumour progression rather than tumour initiation and, further, that the presence of LOH on 16q is associated with a poor prognosis (Grundy et al, 1994). It should be noted that, in the kidney showing nephroblastomatosis, the tissue sampled was from the immediate vicinity of the tumour, which raises the possibility of sample contamination (Austruy et al, 1995).

Wiedemann–Beckwith syndrome (WBS) is considered to be a Wilms' tumour predisposition syndrome (Wiedemann 1983), and has been associated with chromosome region 11p15 by linkage studies in familial cases and the presence of cytogenetic abnormalities involving this region (Koufos et al, 1989; Ping et al, 1989; Weksberg et al, 1993) in patients with WBS. Recently, chromosome 16 has been implicated in this syndrome in two patients (Weksberg et al, 1993; Newsham et al, 1995) and in the Wilms' tumour of a patient with WBS (Austruy et al, 1995). Both of these WBS patients had constitutional translocations derived from phenotypically normal mothers. The first (Weksberg et al, 1993) carried a t(11;16) (p15.5;q12) and the second (Newsham et al, 1995) a t(11;16) (p15.5;q13). One of the tumours studied by Austray et al (1995) was obtained from a patient with WBS. LOH was apparently restricted to 16q13 in the tumour DNA from this patient and extends over an estimated 8-cM region. This tumour-specific deletion of 16q13 markers provides further evidence for the independent involvement of a gene at 16q13 in Wilms' tumorigenesis. A number of patients with WBS and maternally derived translocations have now been reported (Weksberg et al 1993; Mannens et al 1994) and, although 11p15.5 was always involved, chromosome 16 was not a consistent partner chromosome. Furthermore, of the two WBS patients with an 11;16 translocation, one is alive with no evidence of a Wilms' tumour, the other died in the neonatal period (Weksberg et al, 1993; Newsham et al, 1995).

There is some evidence for an association between cytogenetic/molecular abnormalities on chromosomes 11 and 16 in Wilms' tumours. Two studies have analysed LOH in Wilms' tumours on both 11p and 16q. The initial study found LOH for both chromosomes in only three of nine tumours (Maw et al, 1992). Similar findings were reported by Coppes et al (1992), who observed loss of chromosomal material on chromosomes 11 and 16 in three of six tumours. In our study, four of the six Wilms' tumours showing LOH on 16q had previously been analysed for LOH on chromosome 11 (Wadey et al, 1990). Two of these four tumours showed LOH for markers on both 11 and 16. The other two tumours were not included in the LOH analysis of 11p. The fact that LOH on 16q can occur independently of LOH on 11p possibly suggest that the sequential occurrence of these two events is not required for tumorigenesis. However, LOH does not identify mutations in specific genes and so the relative contribution of genes on 11p and 16q will only be resolved when these genes have been characterized.

The association between LOH on 16q and an adverse outcome is clearly potentially very important for future clinical studies, particularly as patients entering clinical trials in the United Kingdom are randomized between immediate nephrectomy vs percutaneous biopsies before preoperative chemotherapy (C Mitchell, personal communication). In order to evaluate LOH on 16q as a prognostic indicator, larger prospective studies involving all of the patients enrolled into the specific trials would be required. Furthermore, if the same polymorphic markers used in the current NWTS-5 trial are used to determine LOH for 16q in future studies, then the frequency of LOH for 16q will be directly comparable. Although there is increasing evidence that LOH for 16q may help stratify patients into those with biologically favourable or unfavourable disease, this possibility needs to be confirmed by analysis of larger numbers of patients. Confirmation of this relationship would possibly enable 'fine tuning' of available therapies for Wilms' tumour with the possibility of reserving the use of doxorubicin, which is cardiotoxic, for use in those patients with adverse biological and clinicopathological features. These extended studies may also serve to further narrow down the critical region of 16q.

### REFERENCES

Austruy E, Candon S, Henry I, Gyapay G, Tournade M-F, Mannens M, Callen D, Junien C and Jeanpierre C (1995) Characterisation of regions of chromosome 12 and 16 involved in nephroblastoma tumorigenesis. *Genes Chrom Cancer* **14**: 285–294

Beckwith JB, Kiviat NB and Bonadio JF (1990) Nephrogenic rests, nephroblastomatosis, and the pathogenesis of Wilms' tumor. *Pediatr Pathol* **10**: 1–36

Bergerheim USR, Kunimi K, Collins VP and Ekman P (1991) Deletion mapping of chromosomes 8, 10 and 16 in human prostatic carcinoma. *Genes Chrom Cancer* **3**: 215–220

Cavenee, WK, Dryja TP, Phillips RA, Benedict WF, Godbout R, Gallie BL, Murphree AL, Strong LC and White RL (1983) Expression of recessive alleles by chromosomal mechanisms in retinoblastoma. *Nature* **305**: 779–784

Coppes MJ, Bonetta L, Huang A, Hoban P, Chilton-Macneill S, Campbell E, Weksberg R, Yeger H, Reeve AE and Williams BRG (1992) Loss of heterozygosity mapping in Wilms' tumour indicates the involvement of three

*© Cancer Research Campaign 1998*

distinct regions and a limited role for nondisjunction or mitotic recombination. *Genes Chrom Cancer* 5: 326–334

D'angio GJ. Evans A. Breslow N. Beckwith B. Bishop H. Farewell V. Goodwin W. Leape L. Palmer N. Sink SL. Sutow W. Tefft M and Wolff J. (1981) The treatment of Wilms' tumour: results of the second National Wilms' Tumour study. *Cancer* 47: 2302–2311

D'angio GJ. Breslow N. Beckwith JB. Evans A. Baum E. Delorimier A. Fernback D. Hrabovsky E. Jones B. Kelalis P. Othersen HB. Tefft M and Thomas PRM (1989) Treatment of Wilms' tumour: results of the third National Wilms' tumour study. *Cancer* 64: 349–360

Doggett NA. Goodwin LA. Tesmer JG. Meincke LJ. Brece DC. Clark LM. Altherr MR. Ford AA. Chi. H-C. Marrone BL. Longmire JL. Lane SA. Whitmore SA. Lowenstein MG. Sutheralnd RD. Mundt MO. Knill EH. Bruno WJ. Macken CA. Torney DC. WU. J-R. Griffith J. Sutherland GR. Deaven LL. Callen DF and Moyzis RK (1995) An integrated physical map of human chromosome 16. *Nature* 377: 355–365

Dorion-Bonnet. F. Mutalen. S. Holstein I and Longy M. (1995) Allelic imbalance study of 16q in human primary breast carcinimas using microsatellite markers. *Genes Chrom Cancer* 14: 171–181

Douglass EC. Rowe ST. Valentine M. Parham D. Meyer WH and Thompson. EI (*1990*) A second non-random translocation der (16)t(1:16)(q21:q13) in Ewings sarcoma and peripheral neuroectodermal tumour. *Cytogenet Cell Genet* 53: 87–90

Fujimori M. Tokino T. Hino O. Kitagawa T. Imamura T. Okamoto E. Mitsunobu M. Nakagama H. Harada H. Yagura M. Matsubara K and Nakamura Y (1991) Allotype study of hepatocellular carcinoma. *Cancer Res* 51: 89–93

Goorin AM. Chauvenet AR and Perez-atayde AR (1990) Initial congestive cardiac failure six to ten years after doxorubicin therapy for childhood cancer. *J Pediatr* 116: 144

Grundy PE. Telzerow PE. Breslow N. Moksness J. Huff V and Paterson MC (1994) Loss heterozygosity for chromosomes 16q and 1p in Wilms' tumours predicts an adverse outcome. *Cancer Res* 54: 2331–2333

Gyapay G. Morisette J. Vignal A. Dib C. Fizames C. Millasseau P. Marc S. Bernardi G. Lathrop M and Weissenbach J (1994) The 1993–94 Genethon human genetic linkage map. *Nature Genet* 7: 246–299

Kaneko Y. Homma C. Maseki N. Sakurai M and Hata J (1991) Correlation of chromosome abnormalities with histological and clinical features in Wilms' and other childhood renal tumors. *Cancer Res* 51: 5937–5942

Knudson AG and Strong LC (1972) Mutation and cancer: a model for Wilms' tumour of the kidney. *J Natl Cancer Inst* 48: 313–324

Koufos A. Grundy P. Morgan K. Aleck KA. Hadro R. Lampkin BC. Kalbakji A and Cavenee WK (1989) Familial Wiedemann–Beckwith syndrome and a second Wilms' tumour locus both map to 11p15.5. *Am J Hum Genet* 44: 711–719

Kozman HM. Keith TP. Donis-Keller H. White RL. Wissenbach J. Dean M. Vergnaud G. Kidd K. Gusella J. Royle. NJ. Sutherland GR and Mulley JC (1995) The CEPH consortium linkage map of human chromosome 16. *Genomics* 25: 44–58

Mannens M. Hoovers JMN. Redeker E. Verjaal M. Feinberg A. Little P. Boavida M. Coad N. Steenman M. Bliek J. Slater RM. DE Boer EG. John R. Cowel. L JK. Junien C. Henry I. Tomerup N. Niikawa N. Weksberg R. Pueschel SM. Leschot NJ and Westerveld. A (1994) Parental imprinting of human chromosome region 11p15.4–pter involved in the Beckwith–Wiedemann syndrome and various human neoplasia. *Eur J Hum Genet* 2: 3–23

Matthew P. Douglass EC. Jones D. Valentine M. Valentine V. Rowe S and Shapiro DN (1996) Der(16) t (1:16)(q21:q13) in Wilms' tumour: friend or foe. *Med Ped Oncol* 23: 3–7

Maw M. Grundy P. Millow L. Eccles M. Dun R. Smith P. Feinberg A. Law D. Patterson M. Telzerow P. Callem D. Thompson A. Richards R and Reeve A (1992) A third tumour locus on chromosome 16q. *Cancer Res* 52: 3094–3098

Mugneret F. Lizard S. Aurias A and Turc-Carel C (1988) Chromosomes in Ewings sarcoma. II. Non-random additional changes. trisomy 8 and der(16)t(1:16). *Cancer Genet Cytogenet* 32: 239–245

Newsham I. Kindler-Rohrborn A. Daub D and Cavenee W (1995) A constitutional BWS-related t(1:16) chromosome translocation occuring in the same region of chromosome 16 implicated in Wilms' tumour. *Genes Chrom Cancer* 12: 1–7

Park S. Bernard A. Bove K. Sens DA. Hazen-Martin DJ. Garvin AJ and Haber DA (1993) Inactivation of WT1 in nephrogenic rests. genetic precursors to Wilms' tumour. *Nature Genet* 5: 363–367

Ping AJ. Reeve AE. Law DJ. Young MR. Boehnke M and Feinberg AP (1989) Genetic linkage of Beckwith–Wiedemann syndrome to 11p15. *Am J Hum Genet* 44: 720–723

Pritchard J. Imeson J. Barnes J. Cotterill S. Gough D. Marsden HB. Morris-Jones P and Pearson D (1995) Results of the United Kingdom children's cancer study group first Wilms' tumour study. *J Clin Oncol* 13: 124–133

Redeker E. Lip KVD. Bliek J. Spelman F. Kraker JD. Voute PA. Westerveld A and Mannens M (1996) *Allele Loss Patterns in Childhood Kidney Tumours*. University of Amsterdam: Amsterdam

Sato T. Tanigami A. Yamakawa K. Akiyama F. Kasumi F. Sakamoto G and Nakamura Y (1990) Allotype of breast carcinoma: Cumulative allele losses promote tumour progression in primary breast cancer. *Cancer Res* 50: 7184–7189

Slater RM and Mannens M (1992) Cytogenetics and molecular genetics of Wilms' tumor of childhood. *Cancer Genet Cytogenet* 61: 111–121

Solis V. Pritchard J and Cowell JK (1988) Cytogenetic changes in Wilms' tumors. *Cancer Genet Cytogenet* 34: 223–234

Sorenson K. Levitt G. Sebag-Montefiore D. Bull C and Sullivan I (1995) Cardiac function in Wilms' tumour survivors. *J Clin Oncol* 13: 1546–1556

Thomas GA and Raffel C (1991) Loss of heterozygosity on 6q. 16q and 17p in human central nervous system primitive neuroectodermal tumours. *Cancer Res* 51: 639–643

Tsuda H. Zhang W. Shimosato Y. Yokota Y. Terada M. Sugimura. T. Miyamura T and Hirohashi S (1990) Allele loss on chromosome 16 associated with progression of human hepatocellular carcinoma. *Proc Natl Acad Sci USA* 87: 6791–6794

Wadey RB. Pal NP. Buckle B. Yeomans E. Pritchard J and Cowell JK (1990) Loss of heterozygosity in Wilms' tumour involves two distinct regions of chromosome 11. *Oncogene* 5: 901–907

Wang-Wuu S. Soukup S. Bove K. Gotwals B and Lampkin B (1990) Chromosome analysis of 31 Wilms' tumors. *Cancer Res* 50: 2786–2793

Weksberg R. Teshima I. Williams BGR. Greenberg CR. Pueschel SM. Chernos JE. Fowlow SB. Hoyme E. Anderson IJ. Whiteman DAH. Fisher N and Squire J (1993) Molecular characterisation of cytogenetic alterations associated with the Beckwith–Wiedemann syndrome (BWS) phenotype refines the localization and suggests the gene for BWS is imprinted. *Hum Mol Genet* 2: 549–556

Wiedemann HR (1983) Tumours and hemihypertrophy associated with Wiedemann–Beckwith syndrome. *Eur J Pediatr* 141: 129

© Cancer Research Campaign 1998


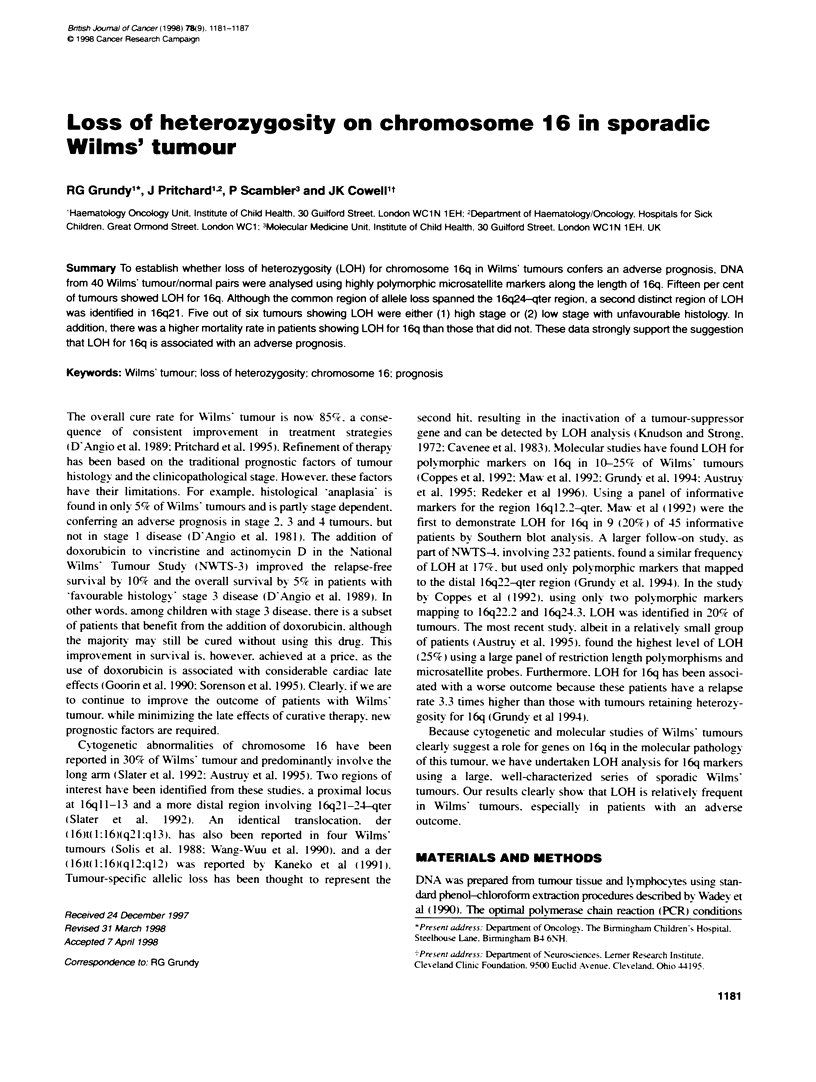

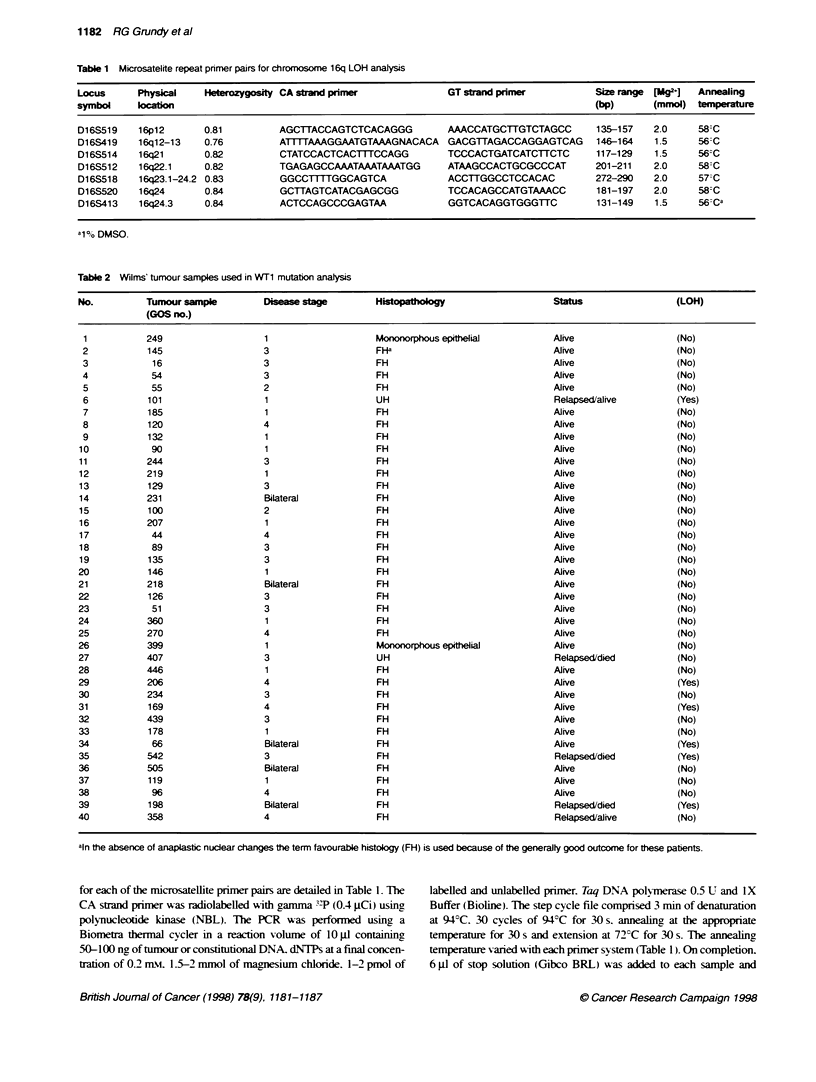

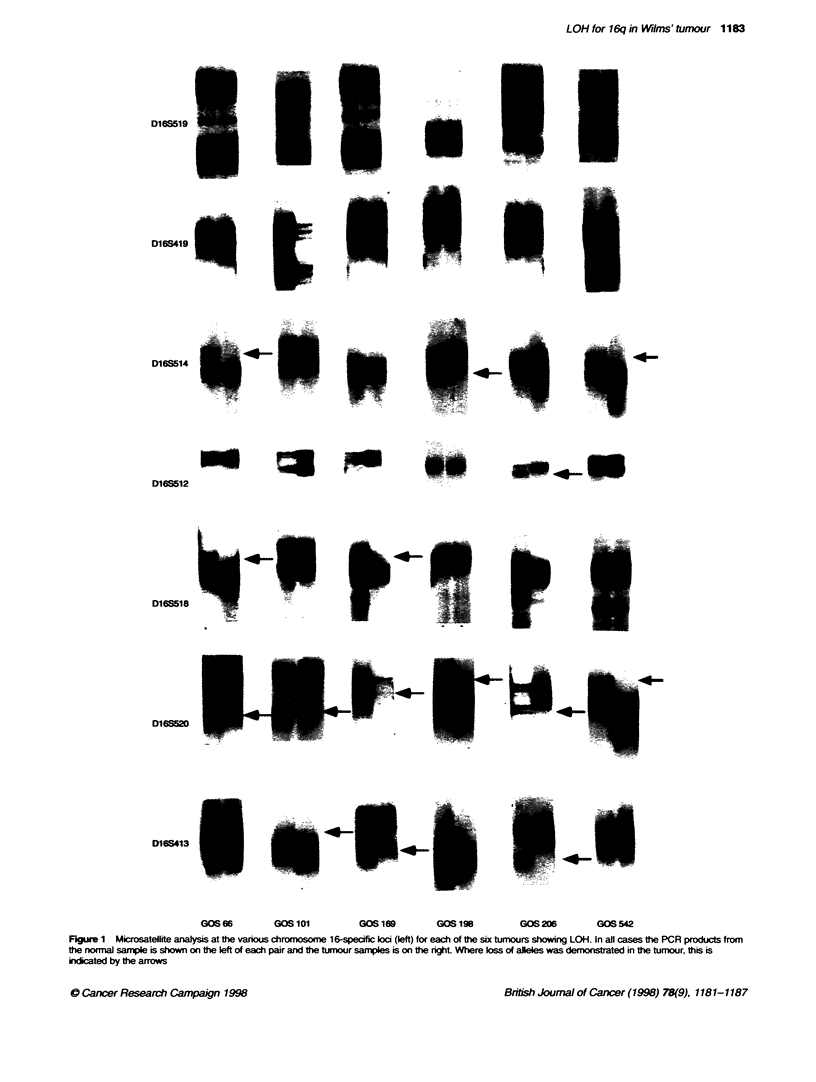

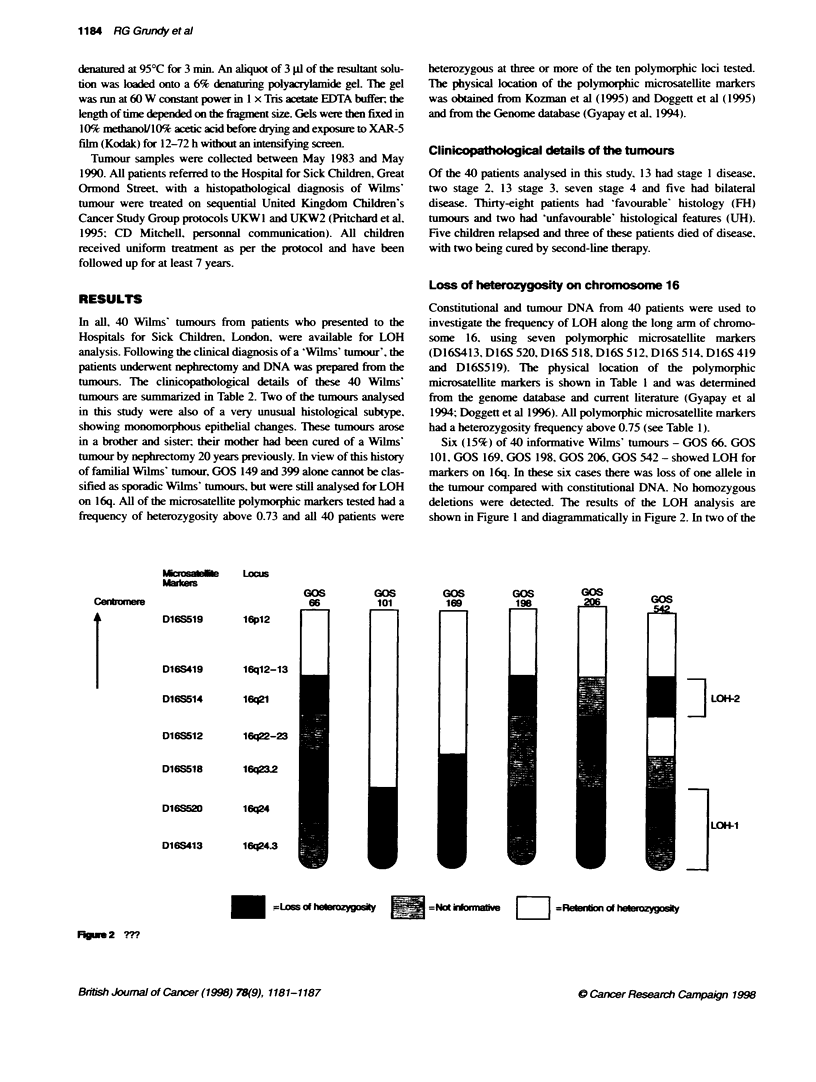

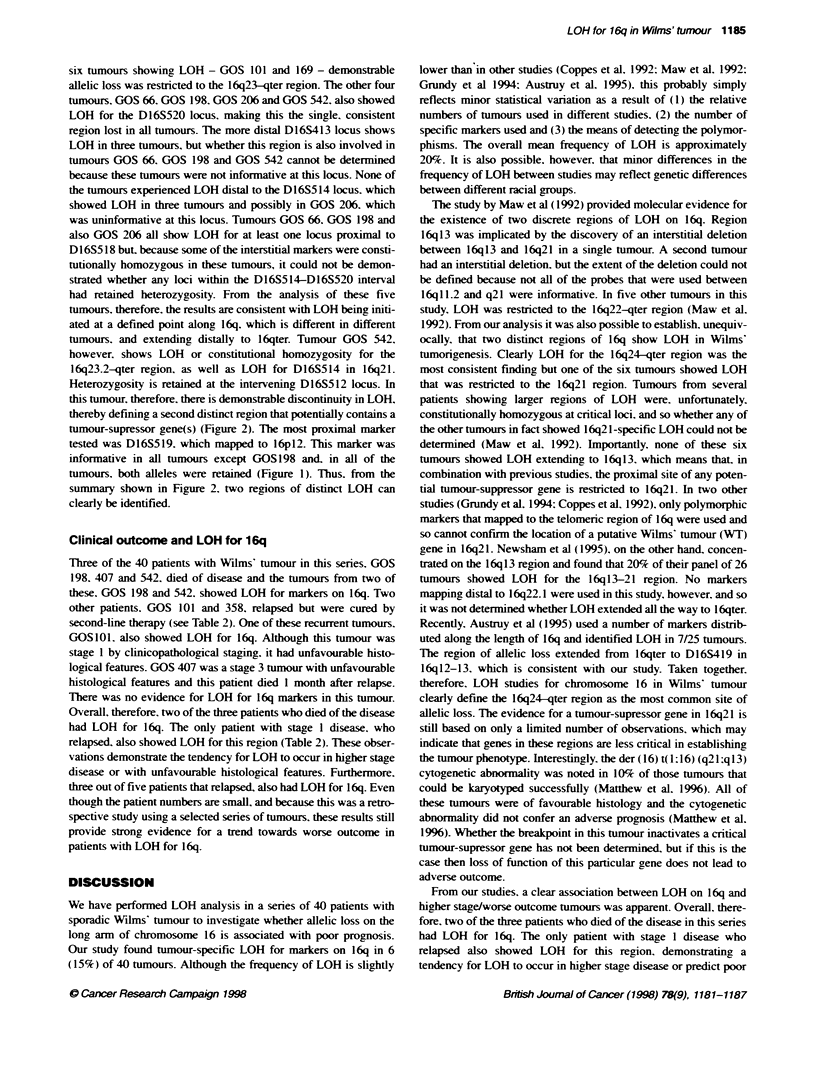

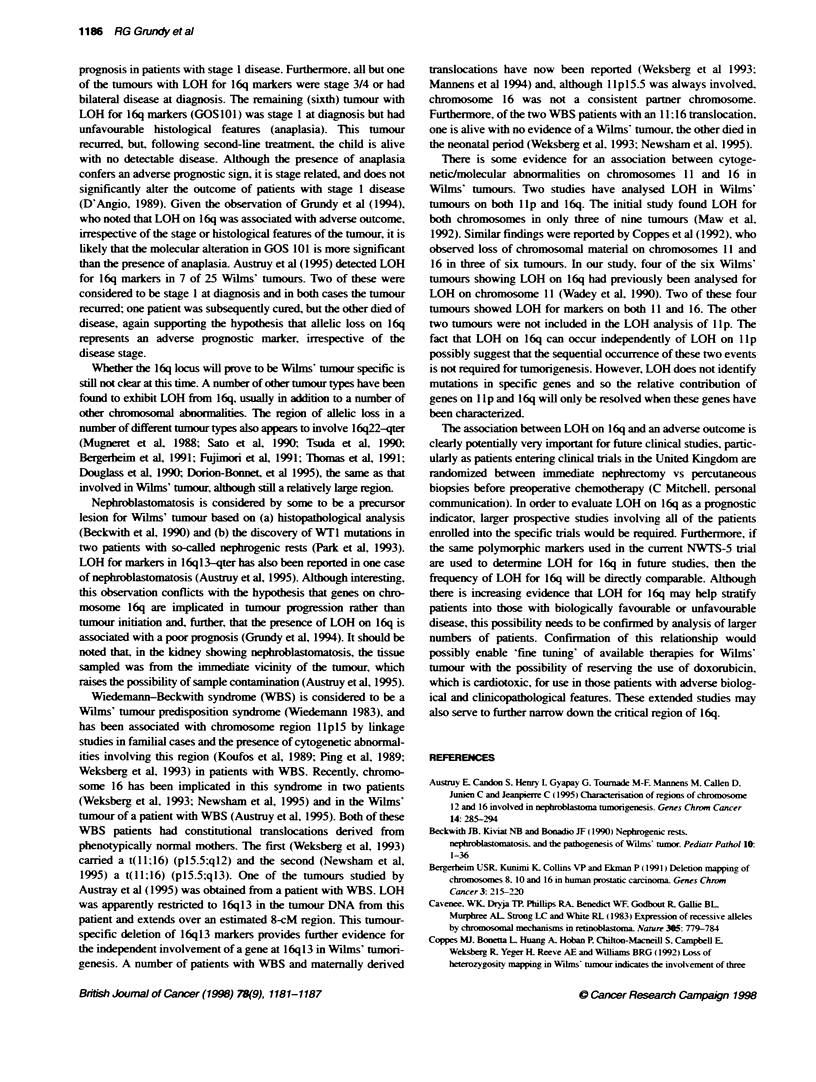

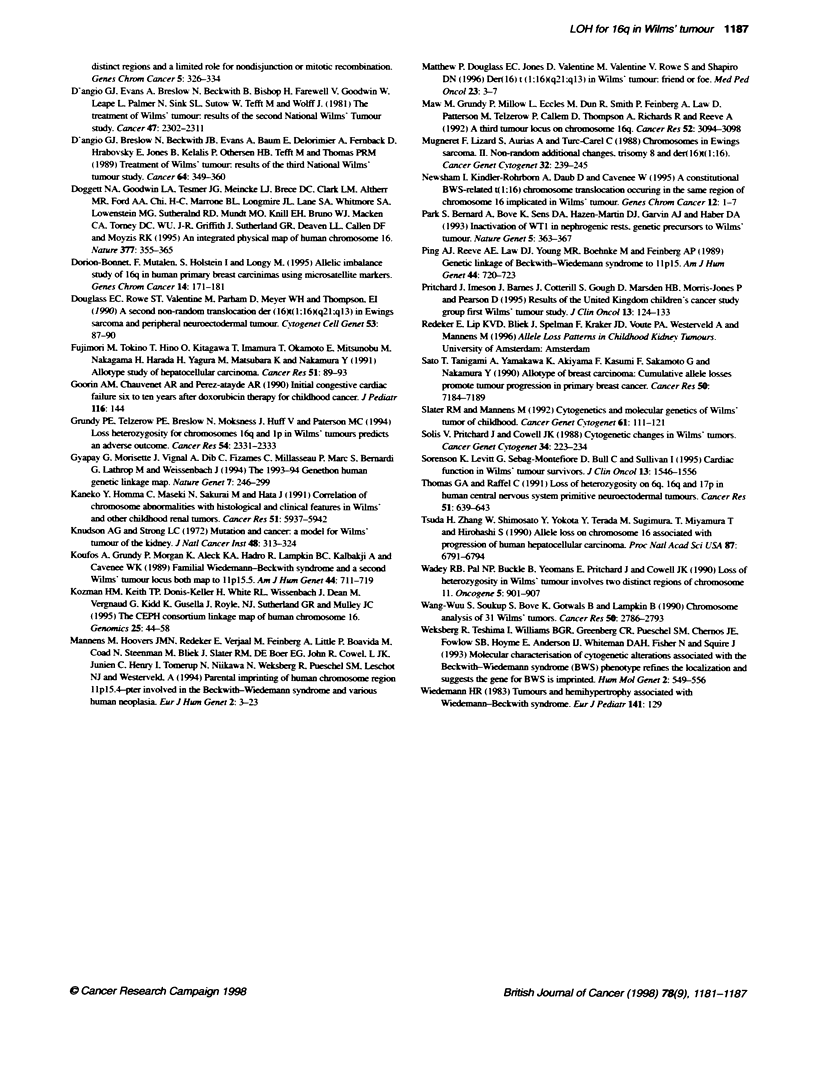


## References

[OCR_00616] Austruy E., Candon S., Henry I., Gyapay G., Tournade M. F., Mannens M., Callen D., Junien C., Jeanpierre C. (1995). Characterization of regions of chromosomes 12 and 16 involved in nephroblastoma tumorigenesis.. Genes Chromosomes Cancer.

[OCR_00620] Beckwith J. B., Kiviat N. B., Bonadio J. F. (1990). Nephrogenic rests, nephroblastomatosis, and the pathogenesis of Wilms' tumor.. Pediatr Pathol.

[OCR_00627] Bergerheim U. S., Kunimi K., Collins V. P., Ekman P. (1991). Deletion mapping of chromosomes 8, 10, and 16 in human prostatic carcinoma.. Genes Chromosomes Cancer.

[OCR_00633] Cavenee W. K., Dryja T. P., Phillips R. A., Benedict W. F., Godbout R., Gallie B. L., Murphree A. L., Strong L. C., White R. L. Expression of recessive alleles by chromosomal mechanisms in retinoblastoma.. Nature.

[OCR_00657] D'Angio G. J., Evans A., Breslow N., Beckwith B., Bishop H., Farewell V., Goodwin W., Leape L., Palmer N., Sinks L. (1981). The treatment of Wilms' tumor: results of the Second National Wilms' Tumor Study.. Cancer.

[OCR_00669] Dorion-Bonnet F., Mautalen S., Hostein I., Longy M. (1995). Allelic imbalance study of 16q in human primary breast carcinomas using microsatellite markers.. Genes Chromosomes Cancer.

[OCR_00674] Douglass E. C., Rowe S. T., Valentine M., Parham D., Meyer W. H., Thompson E. I. (1990). A second nonrandom translocation, der(16)t(1;16)(q21;q13), in Ewing sarcoma and peripheral neuroectodermal tumor.. Cytogenet Cell Genet.

[OCR_00683] Fujimori M., Tokino T., Hino O., Kitagawa T., Imamura T., Okamoto E., Mitsunobu M., Ishikawa T., Nakagama H., Harada H. (1991). Allelotype study of primary hepatocellular carcinoma.. Cancer Res.

[OCR_00687] Goorin A. M., Chauvenet A. R., Perez-Atayde A. R., Cruz J., McKone R., Lipshultz S. E. (1990). Initial congestive heart failure, six to ten years after doxorubicin chemotherapy for childhood cancer.. J Pediatr.

[OCR_00690] Grundy P. E., Telzerow P. E., Breslow N., Moksness J., Huff V., Paterson M. C. (1994). Loss of heterozygosity for chromosomes 16q and 1p in Wilms' tumors predicts an adverse outcome.. Cancer Res.

[OCR_00695] Gyapay G., Morissette J., Vignal A., Dib C., Fizames C., Millasseau P., Marc S., Bernardi G., Lathrop M., Weissenbach J. (1994). The 1993-94 Généthon human genetic linkage map.. Nat Genet.

[OCR_00700] Kaneko Y., Homma C., Maseki N., Sakurai M., Hata J. (1991). Correlation of chromosome abnormalities with histological and clinical features in Wilms' and other childhood renal tumors.. Cancer Res.

[OCR_00705] Knudson A. G., Strong L. C. (1972). Mutation and cancer: a model for Wilms' tumor of the kidney.. J Natl Cancer Inst.

[OCR_00711] Koufos A., Grundy P., Morgan K., Aleck K. A., Hadro T., Lampkin B. C., Kalbakji A., Cavenee W. K. (1989). Familial Wiedemann-Beckwith syndrome and a second Wilms tumor locus both map to 11p15.5.. Am J Hum Genet.

[OCR_00716] Kozman H. M., Keith T. P., Donis-Keller H., White R. L., Weissenbach J., Dean M., Vergnaud G., Kidd K., Gusella J., Royle N. J. (1995). The CEPH consortium linkage map of human chromosome 16.. Genomics.

[OCR_00738] Mugneret F., Lizard S., Aurias A., Turc-Carel C. (1988). Chromosomes in Ewing's sarcoma. II. Nonrandom additional changes, trisomy 8 and der(16)t(1;16).. Cancer Genet Cytogenet.

[OCR_00749] Park S., Bernard A., Bove K. E., Sens D. A., Hazen-Martin D. J., Garvin A. J., Haber D. A. (1993). Inactivation of WT1 in nephrogenic rests, genetic precursors to Wilms' tumour.. Nat Genet.

[OCR_00754] Ping A. J., Reeve A. E., Law D. J., Young M. R., Boehnke M., Feinberg A. P. (1989). Genetic linkage of Beckwith-Wiedemann syndrome to 11p15.. Am J Hum Genet.

[OCR_00757] Pritchard J., Imeson J., Barnes J., Cotterill S., Gough D., Marsden H. B., Morris-Jones P., Pearson D. (1995). Results of the United Kingdom Children's Cancer Study Group first Wilms' Tumor Study.. J Clin Oncol.

[OCR_00769] Sato T., Tanigami A., Yamakawa K., Akiyama F., Kasumi F., Sakamoto G., Nakamura Y. (1990). Allelotype of breast cancer: cumulative allele losses promote tumor progression in primary breast cancer.. Cancer Res.

[OCR_00802] Sheng W. W., Soukup S., Bove K., Gotwals B., Lampkin B. (1990). Chromosome analysis of 31 Wilms' tumors.. Cancer Res.

[OCR_00775] Slater R. M., Mannens M. M. (1992). Cytogenetics and molecular genetics of Wilms' tumor of childhood.. Cancer Genet Cytogenet.

[OCR_00779] Solis V., Pritchard J., Cowell J. K. (1988). Cytogenetic changes in Wilms' tumors.. Cancer Genet Cytogenet.

[OCR_00781] Sorensen K., Levitt G., Sebag-Montefiore D., Bull C., Sullivan I. (1995). Cardiac function in Wilms' tumor survivors.. J Clin Oncol.

[OCR_00787] Thomas G. A., Raffel C. (1991). Loss of heterozygosity on 6q, 16q, and 17p in human central nervous system primitive neuroectodermal tumors.. Cancer Res.

[OCR_00790] Tsuda H., Zhang W. D., Shimosato Y., Yokota J., Terada M., Sugimura T., Miyamura T., Hirohashi S. (1990). Allele loss on chromosome 16 associated with progression of human hepatocellular carcinoma.. Proc Natl Acad Sci U S A.

[OCR_00799] Wadey R. B., Pal N., Buckle B., Yeomans E., Pritchard J., Cowell J. K. (1990). Loss of heterozygosity in Wilms' tumour involves two distinct regions of chromosome 11.. Oncogene.

[OCR_00806] Weksberg R., Teshima I., Williams B. R., Greenberg C. R., Pueschel S. M., Chernos J. E., Fowlow S. B., Hoyme E., Anderson I. J., Whiteman D. A. (1993). Molecular characterization of cytogenetic alterations associated with the Beckwith-Wiedemann syndrome (BWS) phenotype refines the localization and suggests the gene for BWS is imprinted.. Hum Mol Genet.

